# Plasma zinc level during acute gastroenteritis

**DOI:** 10.1186/1471-2334-14-S7-P53

**Published:** 2014-10-15

**Authors:** Nicoleta Negruț

**Affiliations:** 1 Faculty of Medicine and Pharmacy, University of Oradea, Romania

## Background

Gastroenteritis may result in loss of zinc by feces, decreased tissue levels of zinc and negative zinc balance in children.

## Methods

The first aim of the study was to determine the plasma zinc level on admission in the hospital and 10 days after the recovery from acute gastroenteritis, in children aged 0-3 years, from the region of Bihor, Romania. Second aim of the study was to analysis the plasma zinc level according to the etiology of gastroenteritis. Zinc sulfate (10-20 mg daily, according to age) was given to the patients in the study group, for 10 days. The colorimetric method with Br-PAPS final point (CV% 0.98%-4.64%) was used for the determination of the zinc level. The program IBM SPSS statistics version 22 was used for analysis of the data.

## Results

During three years (2009-2011), 103 children with acute gastroenteritis were enrolled in the study. The mean plasma zinc level in the 10^th^ day versus day 0, increased in the study group (n=53) (14.59±2.55 μmol/L versus 15.66±3.98 μmol/L, p=0.049, Student’s test) and decreased in the control group (n=50) (15.08±3.28 μmol/L versus 13.59±3.02 μmol/L, p=0.041, Student’s test). In the day 0, there were no significant differences between plasma zinc level in children with bacterial gastroenteritis compared with viral gastroenteritis (13.97±2.52 μmol/L versus 14.08±2.19 μmol/L, p=0.911, Student’s t test).

## Conclusion

Plasma zinc level decreased after 10 days of acute gastroenteritis. The etiologic agent of diarrhea did not influence the plasma level of zinc.

